# Theme and Variations in the Evolutionary Pathways to Virulence of an RNA Plant Virus Species

**DOI:** 10.1371/journal.ppat.0030180

**Published:** 2007-11-23

**Authors:** Agnès Pinel-Galzi, Mbolarinosy Rakotomalala, Emmanuel Sangu, Fatogoma Sorho, Zakaria Kanyeka, Oumar Traoré, Drissa Sérémé, Nils Poulicard, Yvonne Rabenantoandro, Yacouba Séré, Gnissa Konaté, Alain Ghesquière, Eugénie Hébrard, Denis Fargette

**Affiliations:** 1 Institut de Recherche pour le Développement (IRD), UMR RPB, Montpellier, France; 2 Centre National de la Recherche Appliquée au Développement Rural (FOFIFA), Mahajanga, Madagascar; 3 Botany Department, Dar es Salaam University, Dar es Salaam, Tanzania; 4 Africa Rice Center (WARDA), Cotonou, Bénin; 5 Institut de l'Environnement et de Recherches Agricoles (INERA), Laboratoire de Biotechnologie et de Virologie Végétale, Kamboinsé, Ouagadougou, Burkina Faso; 6 Centre National de la Recherche Appliquée au Développement Rural (FOFIFA), Antananarivo, Madagascar; 7 Institut de Recherche pour le Développement (IRD), UMR GDP, Montpellier, France; The Pennsylvania State University, United States of America

## Abstract

The diversity of a highly variable RNA plant virus was considered to determine the range of virulence substitutions, the evolutionary pathways to virulence, and whether intraspecific diversity modulates virulence pathways and propensity. In all, 114 isolates representative of the genetic and geographic diversity of *Rice yellow mottle virus* (RYMV) in Africa were inoculated to several cultivars with eIF(iso)4G-mediated *Rymv1-2* resistance. Altogether, 41 virulent variants generated from ten wild isolates were analyzed. Nonconservative amino acid replacements at five positions located within a stretch of 15 codons in the central region of the 79-aa-long protein VPg were associated with virulence. Virulence substitutions were fixed predominantly at codon 48 in most strains, whatever the host genetic background or the experimental conditions. There were one major and two isolate-specific mutational pathways conferring virulence at codon 48. In the prevalent mutational pathway I, arginine (AGA) was successively displaced by glycine (GGA) and glutamic acid (GAA). Substitutions in the other virulence codons were displaced when E48 was fixed. In the isolate-specific mutational pathway II, isoleucine (ATA) emerged and often later coexisted with valine (GTA). In mutational pathway III, arginine, with the specific S2/S3 strain codon usage AGG, was displaced by tryptophane (TGG). Mutational pathway I never arose in the widely spread West African S2/S3 strain because G48 was not infectious in the S2/S3 genetic context. Strain S2/S3 least frequently overcame resistance, whereas two geographically localized variants of the strain S4 had a high propensity to virulence. Codons 49 and 26 of the VPg, under diversifying selection, are candidate positions in modulating the genetic barriers to virulence. The theme and variations in the evolutionary pathways to virulence of RYMV illustrates the extent of parallel evolution within a highly variable RNA plant virus species.

## Author Summary

Parallel changes in independently evolving lineages are important, but their contribution to pathogen evolution has not been assessed at the species level. We investigated the extent of phenotypic and genotypic parallel evolution in a highly variable RNA plant virus species, *Rice yellow mottle virus* (RYMV). Isolates representative of the genetic and geographic diversity of RYMV in Africa were inoculated to several rice cultivars with eIF(iso)4G-mediated *Rymv1-2* resistance. The theme and variations in the evolutionary pathways to gain virulence found in the VPg of RYMV illustrate the frequency of parallel evolution. The repeated occurrence of the R48E substitution in the VPg of most strains, whatever the *Rymv1-2* background and plant growth conditions, showed the specificity of parallel evolution that operated through the same pathway, locus, and mutation. The frequency and specificity of parallel mutations indicate, respectively, that RYMV is able to rapidly explore the adaptive landscape, fixing favorable mutations to virulence, and that there are a limited number of pathways across the adaptive landscape. Our results provide insights into the ways an RNA virus species explores the adaptive landscape and into the constraints restricting the number of mutational pathways.

## Introduction

Parallel evolution is the evolution of similar or identical features independently in related lineages when subjected to similar selection pressures [1,2]. Parallel evolution has been reported extensively both in natural isolates and in experimental populations of many microbes, most often viruses [2–4], but also bacteria [5], yeast [6], and protozoa [7]. With viruses, similar amino acid replacements often occurred in immune or antiviral escape variants. This is usually interpreted as the fixation of a mutation with a beneficial effect. However, differences in founding genotypes may result in divergent evolutionary trajectories [5]. So, patterns of adaptation to selective constraints may also be dependent on intraspecific polymorphisms. This is well documented for HIV resistance to antiretroviral agents, where pathways of viral evolution towards drug resistance may proceed through distinct steps and at different rates among different HIV subtypes [8,9]. The objective of this article is to assess the extent of parallel evolution in a highly variable RNA plant virus species. Then, the diversity of *Rice yellow mottle virus* (RYMV) was considered to determine the range of virulence substitutions, to reconstruct the evolutionary pathways to virulence, and to test whether intraspecific diversity modulates virulence pathways and propensity.

The breakdown of host plant resistance is a well-documented case of virus adaptation [10]. Translation initiation factors of the eIF4E and eIF4G families are major determinants of recessive resistance to plant viruses [11,12]. Several studies showed that one or a few amino acid substitutions in the genome-linked viral protein VPg are independently responsible for virulence (see [12] for a recent review). In none of these studies however, was the diversity of the virus species considered. Furthermore, no longitudinal studies (samples collected from the same host plant at sequential times) have been conducted to unravel the accumulation and interplay of mutations associated with virulence over time. RYMV is an appropriate model to address these issues: RYMV is a highly variable virus species (up to 11% in nucleotide [nt] difference in the full genome) [13], an eIF(iso)4G-mediated resistance was identified in rice [14], and the viral genome-linked protein (VPg) of RYMV was determined as the virulence factor [15].

RYMV of the genus *Sobemovirus* [16] occurs in all rice-growing African countries, where it causes heavy yield losses [17]. Its genome harbors four open reading frames (ORFs) [18]. ORF1, which is located at the 5′ end of the genome, encodes the protein P1 involved in virus movement and gene silencing. ORF2, which encodes the central polyprotein, has two overlapping ORFs. ORF2a encodes a serine protease and the VPg. ORF2b, which is translated through a −1 ribosomal frameshift mechanism as a fusion protein, encodes the RNA-dependent RNA polymerase. The coat protein is expressed by a subgenomic RNA at the 3′ end of the genome. RYMV is transmitted by contact during cultural practices and by animal vectors, mainly beetle species (Coleoptera) of the Chrysomelidae family.

Very few rice varieties are resistant to RYMV. The highest level of resistance, characterized by an undetectable virus titer in ELISA tests and the absence of symptoms, is provided by *Oryza sativa indica* cv Gigante and a few Oryza glaberrima cultivars of the Tog series [19,20]. Recently, another highly resistant *indica* cultivar, named Bekarosaka, was found in Madagascar [21]. Resistance is controlled by the recessive gene *Rymv1* [19], which maps on chromosome 4 [22] and encodes the translation initiation factor eIF(iso)4G [14]. Three alleles of *Rymv1* were identified, one in the O. sativa cvs Gigante and Bekarosaka (allele *Rymv1-2*), and two in the O. glaberrima accessions Tog5681 (*Rymv1-3*) and Tog5672 (*Rymv1-4*). Compared to susceptible varieties (allele *Rymv1-1*), the *Rymv1-2* resistance allele is characterized by one amino acid substitution in the central domain of the eIF(iso)4G gene [14]. *Rymv1-2* was introgressed into widely grown *indica* cultivars that are now propagated in the fields.

In this paper, we defined virulence as the genetic ability of a pathogen to overcome genetically determined resistance and to cause a compatible interaction leading to disease [23,24]. Avirulence is the antonym of virulence. With RYMV, virulence is characterized by pronounced and generalized symptoms, full systemicity, high virus titer, and high mechanical transmissibility to resistant plants. The high resistance of cv Gigante was effective against a range of isolates of different RYMV strains [19]. Recently, however, the resistance of Gigante was overcome by isolates of several geographic origins [25–27]. Sequencing one such isolate revealed that a single substitution in the VPg specifically differentiated the wild avirulent type from the evolved virulent variant. Directed mutagenesis indicated that this substitution conferred the virulence [15]. This work, carried out with a single isolate, is extended here to a range of isolates of the main strains. Isolates representative of the genetic and geographic diversity of RYMV in Africa were inoculated to *Rymv1-2*-resistant accessions made of cultivars Gigante and Bekarosaka and of four nearly isogenic lines (NILs). The accumulation and the interplay over time of mutations associated with virulence were followed. Altogether, we found one theme, yet several isolate- or strain-specific variations, in the evolutionary pathways to virulence of RYMV.

## Results

### A Narrow Spectrum of Substitutions Associated with Virulence

**Figure 1:**
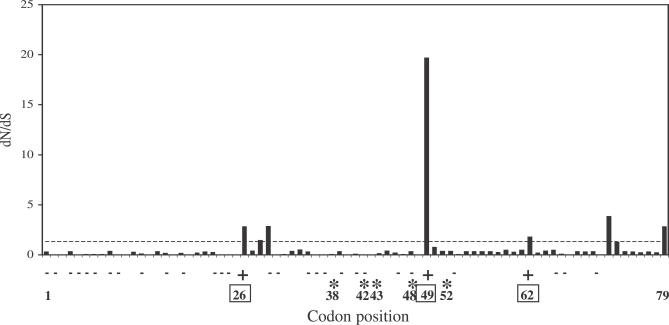
dN/dS at Each Codon of the VPg The dN/dS ratio at each of the 79 codons of the VPg was estimated from a corpus of 59 isolates representative of the genetic and geographic diversity of RYMV. The dotted line indicates the value dN/dS = 1. Codons under diversifying selection (dN/dS > 1) are indicated with a “+” and the codon position is boxed. Codons under negative selection pressure (dN/dS < 1) are underlined with a “-”. Amino acid changes associated with *Rymv1-2* virulence are indicated with a “*” above the codon position.

A total of 22 isolates representative of the geographic and genetic diversity of RYMV was fully sequenced. The dN/dS value of the VPg was lower than that of the P1 and of the coat protein (0.05 versus 0.15 and 0.14, respectively) and similar to that of ORF2b coding the polymerase (0.04). We sequenced the VPg of an additional set of 37 wild avirulent isolates collected in the fields. Analysis of the 59 wild isolates revealed that 17 of the 79 amino acids of the VPg (22%) varied naturally in wild-type isolates (both within and between strains). A total of 29 codons was under conservative selection pressure, most of them in the N-terminal half of the VPg (Figure 1). Only three codons were under positive selection with a Bayes factor for positive selection higher than 100. Codon 49 was under high diversifying selection as apparent from both the REL and IFEL methods, with dN/dS = 20. Codon 26, and codon 62 adjacent to the sobemovirus WAD conserved motif at positions 64–68, were under moderate diversifying selection with dN/dS = 2.8 and 1.8, respectively, and only significant with the less conservative method REL.

**Figure 2:**
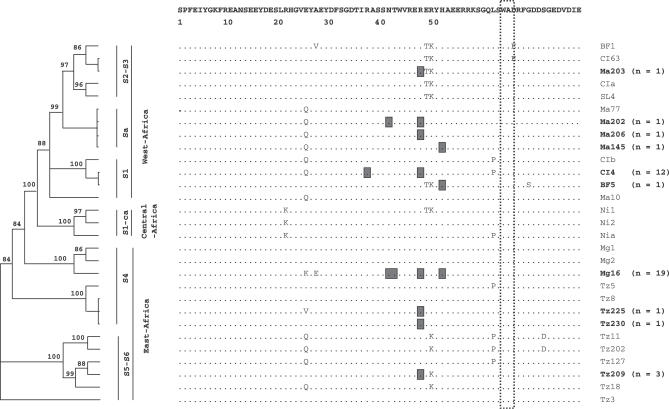
Positions of the Amino Acid Changes Associated with Virulence in the VPg A total of 41 virulent variants generated by inoculating ten wild isolates (names in bold characters) was analyzed. The number of virulent variants analyzed from each of the ten wild isolates is indicated in brackets after the isolate name (right). The amino acid differences between the sequences of the wild isolates of the VPg and the consensus sequence (at the top) are indicated in plain letters. Positions of the amino acid changes associated with virulence are boxed (in black). They were deduced from comparison between the sequences of the VPg of the wild avirulent and the derived virulent forms of each isolate. The large dotted rectangle indicates the conserved WAD motif of the sobemoviruses. The taxonomic positions of the isolates are inferred from the cladogram of 22 reference isolates reconstructed by maximum likelihood from their full sequence with bootstrap support of the nodes, strain, and geographical origins indicated (left). The seven additional isolates were assigned to the closest reference isolate after phylogenetic analyses of their coat protein.

**Figure 3:**
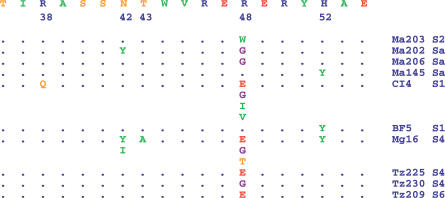
Amino Acid Polymorphism Associated with Virulence in the VPg The consensus sequence of the wild isolates and the positions of the variable codons are indicated at the top of the figure. Amino acid changes associated with virulence were deduced from comparison between the sequences of the VPg of the wild avirulent and the evolved forms of each isolate. The class of the amino acids is indicated with colors: acidic (red), basic (blue), hydrophobic (green), polar (orange), and small (purple).

In all, 114 isolates representative of the main strains of RYMV were inoculated to *Rymv1-2*-resistant accessions. Ten of them (c. 9%) became virulent (Table 1). By large-scale inoculation, we generated several virulent variants from isolates CI4, Mg16, and Tz209, and we analyzed, respectively, 12, 19, and three of them. From each of the seven other isolates, only one virulent variant was studied. Altogether, 41 virulent variants were analyzed. Amino acid changes associated with virulence were deduced from comparison between the sequences of the VPg of the wild avirulent and of the evolved virulent forms of each isolate. With three isolates (Ma145, Tz225, and Tz230), however, only the VPg of the evolved virulent form was sequenced, and putative changes associated with virulence were assessed by comparison with the range of sequences of the wild avirulent isolates. Virulence was always associated with non-synonymous changes. They were located within a stretch of 15 codons in the central domain of the 79-aa-long VPg at codons 38, 42, 43, 48, and 52 (Figure 2). The amino acids associated with virulence were never found in the 59 wild avirulent isolates. Codon 43 was under neutral evolution, and codons 42 and 48 were under conservative selection, whereas codons 38 and 52 were strictly conserved in the avirulent wild isolates (Figure 1). Substitutions associated with virulence always involved a change of biochemical class (Figure 3). Most substitutions were transitions (Table 2). The transversion/transition ratio of 0.15 calculated from the changes associated with virulence was similar to that of 0.13 (+/- 0.03) estimated by maximum likelihood from the corpus of 59 wild isolates.

**Table 1:**
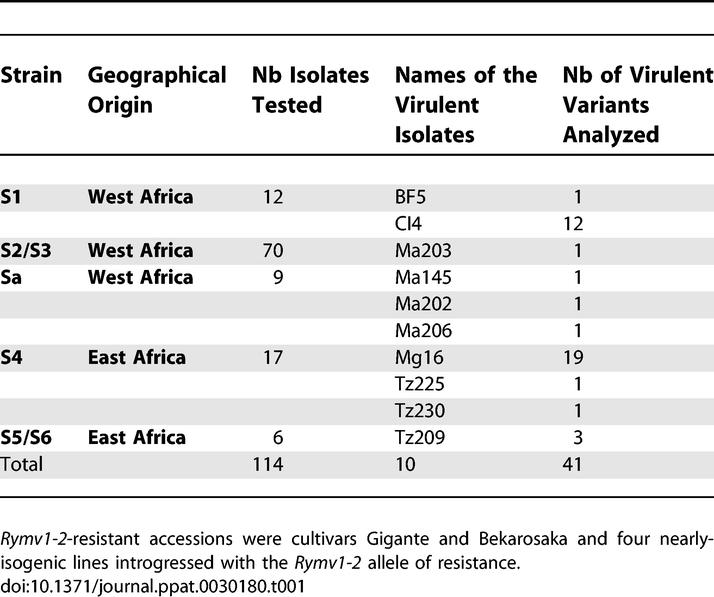
Origin of the Isolates and Number of Virulent Variants Generated by Inoculation on *Rymv1-2* Resistant-Accessions

**Table 2:**
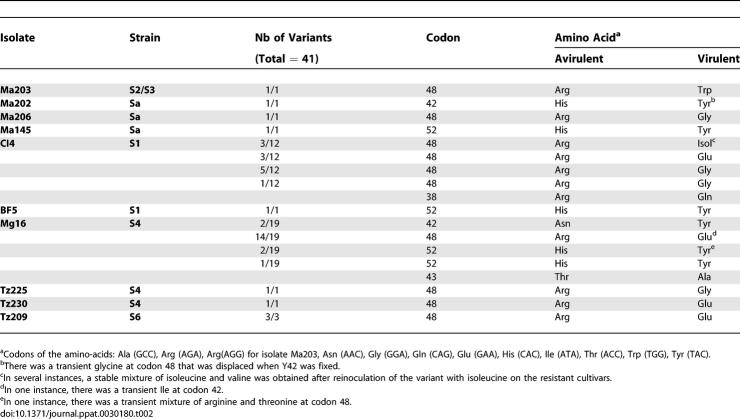
Substitutions Associated to Virulence in the VPg of 41 Virulent Variants Generated by Inoculation of Ten Wild Isolates to *Rymv1-2*-Resistant Cultivars

Substitutions associated with virulence were fixed mostly at codon 48 (33/41), and to a lesser extent at codons 52 (6/41) and 42 (2/41) (Table 2). Changes at codon 48 occurred in all strains, and changes at codon 52 and 42 were found in several strains (Figure 2). Variation was polymorphic at codon 48 with six types of substitutions of the wild arginine (Figure 3). Glycine and glutamic acid substitutions were the most frequent, isoleucine and tryptophane were isolate-specific, and valine and threonine were transient (Table 2). At codon 42, the wild asparagine was displaced by tyrosine and also transiently by isoleucine. Changes at the other codons were monomorphic. Overall, this indicated that despite the large diversity of isolates assessed, virulence was associated with a restricted number of substitutions within the central region of the VPg, codon 48 being overrepresented, and amino acids G48 and E48 the most frequent.

### Three Mutational Pathways to Virulence at Codon 48

**Figure 4:**
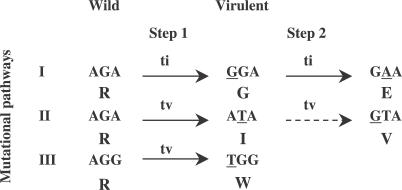
Mutational Pathways to Virulence within Codon 48 of the VPg Mutational pathway I was shared by all strains with the notable exception of strain S2/S3. It involved two successive transitions (ti) from the wild arginine coded by codon AGA. Mutational pathway II was isolate-specific (CI4) and involved two successive transversions (tv). The dotted arrow indicates that V48 arose but did not replace I48. Mutational pathway III was specific for isolate Ma203 of the S2/S3 strain, with the wild arginine coded by codon AGG. It involved a single transversion.

The ultimate sequencing of the VPg of 14 of the 19 virulent variants of isolate Mg16 revealed a glutamic acid (Table 2). The time needed for E48 to emerge ranged from 3 to 7 mo after inoculation (Table S1). Once fixed, E48 remained stable over time. Before E48 fixation, there was either the wild arginine, an R/G mixture, a glycine, or a G/E mixture (Table S1). Changes in codon 48 occurred alongside substitutions in other codons five times, twice with codon 42, twice with codon 52, and once with both (Table S1). In four instances, the changes occurred first in the “alternative” codons 42 or 52, but ultimately E48 was fixed and the alternative substitution was displaced. Overall with isolate Mg16, E48 was the most prevalent virulent variant, and most often emerged after G48 and displaced the other mutants. Similarly, one CI4 virulent variant had a glycine at position 48 and a glutamic acid at a later stage of infection (unpublished data). Altogether, this suggested two successive transitions—from R to G, then G to E—at codon 48 to gain virulence (Figure 4). Glycine and glutamic acid were found in 29 of the 41 virulent variants generated from isolates of all strains with the notable exception of strain S2/S3 (Table 2). Glycine and glutamic acid at codon 48 were fixed in resistant cultivars Gigante and Bekarosaka, and in the four NILs containing the *Rymv1-2* resistance gene. G48 and E48 were generated both in growth chambers and in greenhouse conditions in West and East Africa. Altogether, the two-step R/G/E mutational pathway at codon 48 (named mutational pathway I) was the most prevalent, and occurred in most strains, whatever the genetic background and the experimental conditions.

The two other mutational pathways at codon 48 were isolate-specific. Earlier experiments revealed that the resistance breakdown of cv Gigante after inoculation with isolate CI4 was caused by a R48I substitution in the VPg [15]. Isoleucine emerged three additional times in the present experiments, again after inoculation of isolate CI4 to cv Gigante. The virulent variant CI4 with isoleucine was reinoculated to Gigante. In several instances, sequencing the VPg 5 mo after infection revealed a mixture of isoleucine (ATA) and valine (GTA). The isoleucine-valine mixture was still apparent after re-inoculation of resistant plants. This indicated that valine was stable after emergence, but did not displace isoleucine. The successive R/I/V amino acid emergence suggested a second mutational pathway at codon 48 (named mutational pathway II) gained by two transversions. In all, 70 S2/S3 isolates were inoculated to *Rymv1-2* accessions and more than 1,000 plants were tested. Only one virulent variant emerged from isolate Ma203. Arginine at codon 48, coded by AGG as in most S2/S3 isolates versus AGA in the other strains, was displaced by tryptophane (TGG) (Table 2). This amino acid was never observed in any wild-type isolates or in any of the 40 other virulent isolates. There was no substitution elsewhere on the genome. This one-step mutational pathway (named mutational pathway III) involved a single nucleotide transversion.

### Contrasted Genetic Barriers to Virulence between Strains

The genetic barrier to virulence was defined, by analogy to viral drug resistance [28], as the propensity for the virus to overcome the resistance by developing virulence mutations. Marked differences in virulence propensity were found among isolates and strains of RYMV. Only one isolate of the 70 S2/S3 isolates (1.4%) tested gained virulence. This percentage was significantly lower than that of the other strains (χ^2^ = 9.8, df = 1, *p* = 0.001 in the Yates corrected χ^2^ test), where nine of the 44 isolates (20.5%) overcame the *Rymv1-2* resistance, a percentage similar to the 17.5% estimated earlier using a collection of 280 S1 and Sa isolates from the Sudano-savannah zone [27]. Considering that more large-scale experiments were conducted with S2/S3 isolates (over 1,000 plants were tested in total), the difference between S2/S3 and other strains was still underestimated. Accordingly, the genetic barrier to virulence was higher in S2/S3 than in any other strain.

Failure of the S2/S3 strain to follow mutational pathway I did not reflect its specific codon usage at codon 48, as glycine (GGG) and glutamic acid (GAG) could also be coded by two successive transitions from arginine (AGG). However, strain S2/S3 was the only strain with the motif TK at codons 49 and 50. Other strains had the motifs ER or EK, except some variants of the S1 and S1-ca strains (Figure S1). Three substitutions distinguished the two motifs, two non-synonymous substitutions at codon 49 (nt 145 and 146), and one at codon 50 (nt 149) (Figure S1). (AGG)48 was found when (ACG)49 was fixed, which itself occurred only with (AAG)50. The Pagel correlation test indicated that these changes were coordinated (*p* < 0.01), and the highly conservative concentration changes test further suggested that the changes were concentrated (*p* = 0.07). Altogether, failure of the S2/S3 strain to follow mutational pathway I was associated with the presence of T49. Interestingly, codon 49 was under diversifying selection.

Isolate Mg16 (strain S4), which originated from the northwest of Madagascar, overcame *Rymv1-2* resistance at the exceptional rate of 80%, whereas only 1%–20% of the plants gained virulence in the other isolates, a percentage consistent with that estimated earlier [27]. In one additional survey, 13 other isolates from Madagascar were inoculated to resistant plants. Only isolates Mg11 and Mg35 broke the resistance at a rate as high as that of Mg16 (8/20 and 9/20 infected resistant plants, respectively). As isolate Mg16, they originated from the northwest of Madagascar in the region of Marovoay and had the same genetic determinism of virulence (E48 was fixed in Mg35, whereas there was a G/E mixture at codon 48 or a Y52 substitution in Mg11). Isolates Mg11, Mg16, and Mg35 differed from any other isolates in their VPg by a lysine at codon 26 and a glutamic acid at codon 28 (Figure 2). In another survey, seven out of 54 isolates from 16 sites of eastern Tanzania readily overcome the resistance (unpublished data). Six of them belonged to the S4 strain, and like isolate Tz225, originated from the north of Lake Malawi and had fixed a G48 or E48. They all shared a valine at codon 26, an amino acid never found in any wild or resistance-breaking isolates. Altogether, high virulence propensity in two geographically localized variants of the S4 strain was associated with lysine and valine at codon 26. Interestingly, codon 26 was under diversifying selection.

### Full Sequencing and Directed Mutagenesis

The virulence substitution R48I in the VPg had been identified by comparison of the full sequences of the evolved and the wild isolate CI4, and validated by mutation of the infectious clone CIa [15]. Similarly, a virulent variant from isolate CI4 with G48 was fully sequenced and compared to the wild CI4 isolate. The virulent mutant differed specifically from the wild CI4 by only one substitution in the VPg: R(AGA)48G(GGA). The four other changes, three synonymous (C1547T, T2464C, T3223C) and one non-synonymous (C3395T) substitutions, all located outside the VPg, were frequent in wild avirulent isolates. Full sequencing of this virulent variant at a later stage of infection revealed an E48 in the VPg instead of a G48 and no changes elsewhere in the genome. Subsequently, G48 and E48 were the only candidate substitutions to virulence. The substitutions A1728G and G1729A were introduced by directed mutagenesis into the VPg of the infectious CIa clone to produce a non-synonymous R48E substitution in order to validate the role of E48 in resistance breaking. The mutated clone CIa with E48 was fully infectious in the resistant cultivars Gigante and Bekarosaka. This indicated that E48 caused virulence and that the S2/S3 genetic background of the CIa clone did not interfere with infection. The substitution A1728G was also introduced into the VPg of the infectious clone to produce a non-synonymous R48G. By contrast, the mutated clone with G48 was not infectious either in the susceptible cv IR64 or in the resistant cv Gigante. Doubling the amount of transcript at inoculation failed to induce successful infection. Another attempt, where the infectious clone was mutated to produce G48 with the alternative codon GGA (instead of GGG), also failed to infect either the susceptible cv IR64 or the resistant cv Gigante. At codon 52, the causal role of substitution H52Y in resistance breaking was successfully validated by introduction of the T1740C into the CIa infectious clone.

## Discussion

The theme and variations found in the evolutionary pathways to virulence of RYMV illustrate the extent of parallel evolution within a widely variable RNA plant virus species. In particular, the independent and repeated occurrence of E48 to gain virulence in most strains of RYMV, whatever the *Rymv1-2* genetic background and plant growth conditions, showed a high degree of parallel evolution. The high frequency of parallel mutations during intervals of virulence acquisition indicates both that RYMV is able to rapidly explore the adaptive landscape, fixing favorable mutations to virulence, and that there are a limited number of pathways across the adaptive landscape. Our results provide insights into the ways RYMV explores the adaptive landscape and into the constraints restricting the number of mutational pathways.

RYMV most often gained virulence through two-step mutational pathways between adjacent states: R(AGA) to G(GGA) to E(GAA) (mutational pathway I), and R(AGA) to I(ATA) to V(GTA) (mutational pathway II). In mutational pathway I, glutamic acid arose and replaced glycine, whereas in mutational pathway II valine arose but co-existed with isoleucine. Competitive exclusion of emerging variants also occurred between codons, with substitution E48 displacing alternative virulence substitutions. In a few plant viruses, creation of chimeric viruses or directed mutagenesis of infectious clones showed that two mutations in two different codons were necessary to gain virulence [29]. However, host–plant adaptation, such as virulence through an ordered succession of substitutions, moreover within the same codon, had never been reported. These results suggest that virulence was most often gained through a process of stepwise competitive exclusion of emerging variants. This stepwise and ordered accumulation of substitutions between adjacent states did not support the alternative scenario of a selection of pre-existing virulent isolates within a pool of variants. Consistently, work with cloned infectious proviruses of the simian immunodeficiency virus showed that the *env* gene evolved along similar paths in different individual hosts and that the parallel mutations were generated de novo rather than selected from viral quasispecies [30].

The critical role of virus polymorphism in shaping evolutionary pathways was most apparent with strain S2/S3. Virulence mutational pathway III with tryptophane at codon 48 was specific to isolate Ma203 of the S2/S3 strain. In isolate Ma203, as in most S2/S3 isolates, arginine at position 48 was coded by AGG instead of AGA in the other strains. Then, tryptophane (TGG) can be coded by a single substitution from AGG codon, whereas two substitutions are necessary from AGA. This difference in codon usage likely explains why mutational pathway III was not followed except in strain S2/S3. Moreover, strain S2/S3 did not follow the major mutational pathway I. G48 was never observed experimentally in S2/S3 isolates. The S3 clone CIa mutated with G48 was not infectious. As G48 conferred virulence to *Rymv1-2*-resistant lines in isolates of any other strains, G48 may be unfit in the S2/S3 genetic context. This possibly blocked the major mutational pathway to virulence as simultaneous double mutation from arginine (AGG) to glutamic acid (GAG) was most unlikely. Similarly, PVX variants with single mutations, intermediate in production of doubly-mutated resistance-breaking isolates, were counter-selected, explaining the durability of the Rx1 resistance [31]. Consistently, from a given influenza A virus hemagglutinin (HA) sequence, several mutations were required to yield an antigenically distinct HA, but little or no fitness advantage was conferred by any subset of these mutations [4].

The extent of parallel evolution to gain virulence reflects the specificity of the VPg–eIF(iso)4G relationship and the restricted number of ways to restore compatible interactions between RYMV and the *Rymv1-2* resistance gene. Although the diversity of the highly variable RYMV was considered, the substitutions conferring virulence were localized within a 15-aa-long stretch within the central domain of the VPg. This suggested that this domain (aa 38 to 52), especially position 48, played a critical role in the interaction with the eIF(iso)4G protein involved in *Rymv1-2* resistance. Similarly, the central domain of the VPg of several potyviruses was involved in the interaction with host translation initiation factors [32]. Glutamic acid substitution at codon 48 was the final stage of the prevalent mutational pathway I. An E/K substitution at codon 309 of the eIF(iso)4G gene in rice conferred resistance to RYMV [14]. Arginine and lysine are basic amino acids, whereas glutamic acid is acidic. Accordingly, the E48–K309 interaction between the virulent isolate and the resistant rice would restore more efficiently than any other variants the charge complementarity of the R48–E309 interaction between the avirulent isolate and the susceptible rice. This coordinated pattern of substitutions between the resistance gene and the virulence determinant suggested a direct binding between position 48 of the VPg and position 309 of the eIF(iso)4G that should be tested experimentally.

A high genetic barrier to virulence of strain S2/S3 was associated with threonine at position 49. High propensity to virulence of localized geographical S4 variants in northwestern Madagascar and western Tanzania was linked, respectively, to lysine and valine at position 26. Codons 49 and 26 of the VPg, candidate positions in modulating the genetic barrier to virulence, were both under diversifying selection. Then, they played a critical role in virus evolution, possibly through virulence. Similarly, with the human rhinovirus, a large proportion of diversifying residues was found in the vicinity of domains influencing the RNA/VPg primer binding [33], and sites of the VPg of some plant viruses under positive selection were also involved in virulence [34,35]. Possibly, the adjacent T49 in the S2/S3 strain, instead of E49 in the other strains, altered the ability of G48 to bind site 309 of the eIF(iso)4G gene. This functional dependency between adjacent codons, a possible case of sign epistasy [36], was also observed with HIV-1 [37].

Control of RYMV in Africa through propagation of *Rymv1-2* resistance was initiated recently. Our results suggest that this strategy is most likely to be successful in the forested parts of West Africa where isolates of the S2/S3 strain with a high genetic barrier to virulence exclusively occurred. By contrast, in several other regions, the resistance is likely to be challenged. In savannah and sahelian regions of West Africa, the proportion of isolates able to overcome the resistance of cv Gigante reached 15% [27]. The proportion of virulent isolates was higher in Central than in West Africa. Our results further introduced the idea of a contrasted geographical distribution of localized variants with a high propensity to virulence in regions with a majority of isolates with a high genetic barrier to virulence. The risk of selection and spread of these variants after propagation of *Rymv1-2* resistance should be assessed.

The strategy of an RNA plant virus such as RYMV to gain virulence against host resistance showed striking parallels with HIV resistance against antiviral treatments. (i) When antiviral therapy fails to be fully suppressive, viral variants with decreased susceptibility to protease inhibitors (PIs) can emerge [38]. Similarly, emergence of virulent RYMV isolates by mutation assumed residual multiplication of the wild isolate in the *Rymv1-2* accessions. Accordingly, low but significant multiplication of wild isolates in resistant rice cultivars was detected in Q-RT-PCR (N. Poulicard, A. Pinel, and E. Hébrard, unpublished results). Subsequently, the combination of a small amount of replication of the wild-type isolate and the strong selection pressure imposed by host plant resistance allows the virulent variant to emerge and to displace the wild type. (ii) The development of resistance to PIs is usually a gradual process, and the development of high levels of resistance usually requires an ordered accumulation of multiple mutations in the viral protein [39]. With RYMV, the stepwise R/G/E and R/I/V substitutions at codon 48 and the displacement of other changes in alternative codons over time also illustrate the gradual process leading to virulence. (iii) Upon PI treatment, differences in baseline polymorphism between HIV-1 subtypes may result in the evolution of drug resistance along distinct mutational pathways, or in differences in the incidence of these specific pathways [8,9,28,40]. Interestingly, synonymous genetic polymorphism between HIV-1 subtypes at key resistance mutations also influenced mutational routes to drug resistance [41]. Similarly, genetic diversity of RYMV—including synonymous polymorphism—affected the mutational pathways and the virulence propensity. (iv) The primary mutations against PIs did not occur in wild-type polymorphism, but developed during the course of antiviral treatment failure [8]. Consistently, RYMV virulence mutations were not found in wild isolates but emerged throughout the process of infection in resistant plants. (v) Additional (novel, minor, secondary) mutations, some of them in the close environment of key virulence mutations, modulated the genetic barrier to the development of drug resistance [38,40,42]. Similarly, amino acids at codons 49 and 26 in the VPg of RYMV were candidate positions to modulate the genetic barrier to virulence among strains and variants. The similarities in the processes of evolutionary changes between RYMV and HIV-1 to gain, respectively, virulence against a host plant resistance and resistance to antiviral treatments illustrate that common mechanisms operate in RNA virus evolution and that similar forces shape the genetic structure of their populations [43].

## Materials and Methods

### Plant material.

The response to RYMV of the *Rymv1-2* allele of resistance was tested in six genetic backgrounds made of two cultivars and four NILs. Cultivars Gigante and Bekarosaka are two *indica* cultivars that share the *Rymv1-2* allele of resistance [21]. Cultivar Bekarosaka originated from Madagascar, whereas cv Gigante is assumed to be from Mozambique. *Rymv1-2* was introgressed into three widely grown susceptible *indica* cultivars (BG90–2, Bouaké189, and IR64), and into one partially resistant *japonica* cultivar (Nipponbare) to derive NILs. Cultivars Gigante and Bekarosaka were challenged with isolates of the major strains, whereas the NILs were inoculated only with isolate CI4 of strain S1. The plants were kept in a growth chamber under 12-h illumination at 120 μEm^−2^s^−1^ of PAR at 28 °C and 90% humidity.

### Virus isolates.

In all, 114 isolates representative of the main strains of RYMV and originating from various regions of Africa were collected on susceptible plants in the fields and inoculated to the *Rymv1-2* accessions: West Africa (strain S1 from savannah, S2/S3 from forest, and Sa from sahelian regions), East Africa (S4, S5/S6), and Madagascar (S4) (Table 1). Strains S2 and S3, earlier considered as distinct, were gathered together (referred to as S2/S3 strain), as more intensive survey revealed a continuum between the two strains. In each experiment, the isolate was inoculated to ten to 20 plants per *Rymv1-2* accession. Larger scale experiments with inoculations of 50–200 plants were also conducted either to generate a virulent isolate from a recalcitrant strain (S2/S3), or to obtain several virulent variants from the same wild isolate (CI4, Mg16, Tz209) in order to assess the intra-isolate spectrum of substitutions associated with virulence. Inoculum was prepared by grinding infected frozen leaves in 0.1 M phosphate buffer (pH 7.2) (0.1g/ml). Extracts were mixed with 600-mesh carborundum and rubbed on leaves of 14-d-old rice seedlings. Symptoms were monitored weekly and virus content was assessed by ELISA over time. Plants were kept up to 13 mo after inoculation. Such a length of time is biologically realistic, even for annual cultivated rice with a growing season of c. 3 mo, as regrowth of infected rice stubble is frequent after harvesting. Isolates that induced high virus content and/or generalized symptoms on *Rymv1-2* accessions were collected.

### Virus sequencing and directed mutagenesis.

A total of 59 isolates from the different geographical regions in Africa and all of the strains of RYMV were collected in the fields (referred to as the wild type) and analyzed, usually after multiplication in the susceptible *indica* cultivar IR64 in greenhouses in order to increase virus content (Table S2). Twenty-two of the 59 isolates were fully sequenced as described previously [18]. The VPg and its 5′ and 3′ neighboring regions of the 37 other isolates were sequenced as done earlier [15]. For phylogenetic purposes, the coat protein of these isolates was also sequenced as described elsewhere [44]. The VPg of virulent variants generated by infection of *Rymv1-2*-resistant accessions (referred to as the evolved virulent variant) from seven of these 59 isolates was sequenced. Complementarily, the VPg of three virulent isolates generated in greenhouse conditions at the experimental sites of the INERA research station of Kamboinsé near Ouagadougou (Burkina Faso, West Africa) and of the Dar Es Salaam University Department of Botany (Tanzania, East Africa)—where temperature, light, and humidity were less controlled than in growth chamber, but closer to field conditions—was studied. The same plants were tested before and after resistance breakdown. The last fully developed leaf was sampled. Altogether, 41 virulent variants were analyzed. Multiple peaks at the same position in a sequencing electrophoregram are currently interpreted as reflecting nucleotide polymorphism [45]. In our experiments, electrophoregrams with distinct double peaks at a position were regarded as indicating a mixture of two nucleotides. Displacement was further suggested if one nucleotide was identified singly at an early stage and the other at a later stage of infection. Full sequence comparison and directed mutagenesis were done as described elsewhere [15,18,46] to validate the role of the major substitutions to virulence.

### Sequence analysis.

The phylogeny of the 22 fully sequenced isolates representative of the geographic and genetic diversity of RYMV was reconstructed by maximum likelihood method with the HKY model [47] using an heuristic search implementing a tree bisection and reconnection swapping algorithm applied in PAUP [48]. The transversion/transition (tv/ti) ratio and the alpha parameter of the gamma distribution of the among-site variation were estimated by maximum likelihood. The bootstrap support of the nodes was estimated by 100 replicates with the full heuristic search. Analysis of the genetic diversity of the VPg was based on 59 isolates collected in the fields representative of the geographic and genetic diversity of RYMV, including the 22 fully sequenced reference isolates. The ratio of non-synonymous (dN) versus synonymous (dS) substitutions of the VPg of the 22 reference isolates was calculated as implemented in DnaSP [49] and compared to ORF1, ORF2a, ORF2a+b, VPg, and ORF4. The dN/dS ratio at individual codons in the VPg was calculated on the corpus of 59 isolates using two maximum likelihood methods, Random Effect Likelihood (REL) and Internal Fixed Effect Likelihood (IFEL) [50,51], to determine on each of the 79 codons whether the selection pressure was conservative (negative) (w < 1), neutral (w = 1), or diversifying (positive) (w > 1). REL is an improved variant of the Nielsen-Yang approach, which allows both dS and dN to vary independently across sites. IFEL is a new likelihood method to fit an independent dN and dS to every site in the context of codon substitution and test whether dN ≠ dS. The analyses were conducted with the VPg sequence (nt 1587–1823; 237 nt), and with the VPg and the flanking regions (nt 1526–2065; 540 nt) in order to increase the statistical power of the tests. The tv/ti ratio in the VPg was estimated from the corpus of 59 isolates by maximum likelihood as implemented in HYPHY. Evidence for correlated evolutionary change in two characters (i.e., nucleotides or amino acids at different positions) was tested by the Pagel correlation test [52] as implemented in Mesquite [53]. The concentration changes test [54] as implemented in MacClade [55] was applied to determine whether changes in one character (the dependent character) are concentrated on branches of a tree that have a particular state of a second character (the independent character).

## Supporting Information

Figure S1 Polymorphism at Codons 48-49-50 of the VPg Top: Distribution of the nucleotides at codons 48-49-50 in the VPg of 59 RYMV isolates representative of the genetic and geographic diversity of RYMV. The E/T substitution in codon 49 is coded by two nucleotide changes at positions 145 (G to A) and 146 (A to C). The R/K substitution at codon 49 is coded by one nucleotide change at position 149 (G to A). The strains and the variants with the TK motif at codons 49 and 50 (instead of the ER motif) are indicated at the top of the figure. Bottom: most parsimonious reconstruction of the change of nucleotides at position 146 of codon 49 along the phylogeny of RYMV.
(76 KB PPT)

Table S1 Substitutions over Time Associated to Virulence in the VPg of Isolate Mg16 (S6) Inoculated to *Rymv1-2*-Resistant Cultivars A total of 19 virulent variants generated from isolate Mg16 and labeled Mg16-1 to Mg16-19 was analyzed. They were inoculated to cv Bekarosaka or Gigante. The last developed leaves of the plants were collected. The VPg of the virulent variants was sequenced at different times, up to 13 months after inoculation. Differences with the VPg of the avirulent wild isolate Mg16 are underlined.
(24 KB XLS)

Table S2 Name, Geographical Origin, Strain, and Accession Numbers of the Isolates of *Rice yellow mottle virus* Whose VPg Was Sequenced In all, 62 isolates were fully or partly sequenced. A total of 59 were wild avirulent isolates collected in the fields. Twenty-two of them were fully sequenced. The VPg of virulent variants derived from seven of them was sequenced. Three evolved virulent isolates generated by inoculation on resistant plants in greenhouses were also studied.
(26 KB XLS)

## Abbreviations

NIL: nearly isogenic line
ORF: open reading frame
PI: protease inhibitor
RYMV: *Rice yellow mottle virus*
